# “How am I going to live?”: exploring barriers to ART adherence among adolescents and young adults living with HIV in Uganda

**DOI:** 10.1186/s12889-018-6048-7

**Published:** 2018-10-04

**Authors:** Sarah MacCarthy, Uzaib Saya, Clare Samba, Josephine Birungi, Stephen Okoboi, Sebastian Linnemayr

**Affiliations:** 1 RAND Corporation, Behavioral and Policy Sciences, 1776 Main Street, Santa Monica, CA USA; 2 Pardee RAND Graduate School, 1776 Main Street, Santa Monica, CA USA; 3 TASO Uganda, Old Mulago Complex, Kampala, P.O.Box 10443, Kampala, Uganda; 4 Old Mulago Complex, Kampala P.O.Box 10443, Kampala, Uganda; 5 Infectious Diseases Institute, Makerere University, Kampala, Uganda; 6 RAND Corporation, Economics, Sociology, and Statistics, 1776 Main Street, Santa Monica, CA USA

**Keywords:** Uganda, Adolescents/youth, HIV/AIDS, ART adherence, HIV infections/prevention & control/transmission, Young adult

## Abstract

**Background:**

Studies from sub-Saharan Africa (SSA) document how barriers to ART adherence present additional complications among adolescents and young adults living with HIV. We qualitatively explored barriers to ART adherence in Uganda among individuals age 14–24 to understand the unique challenges faced by this age group.

**Methods:**

We conducted focus group (FG) discussions with Community Advisory Board members (*n* = 1), health care providers (*n* = 2), and male and female groups of adolescents age 14–17 (*n* = 2) and youth age 18–24 (*n* = 2) in Kampala, Uganda. FGs were transcribed verbatim and translated from Luganda into English. Two investigators independently reviewed all transcripts, developed a detailed codebook, achieved a pooled Cohen’s Kappa of 0.79 and 0.80, and used a directed content analysis to identify key themes.

**Results:**

Four barriers to ART adherence emerged: 1) poverty limited adolescents’ ability to buy food and undercut efforts to become economically independent in their transition from adolescence to adulthood; 2) school attendance limited their privacy, further disrupting ART adherence; 3) family support was unreliable, and youth often struggled with a constant change in guardianship because they had lost their biological parents to HIV. In contrast peer influence, especially among HIV-positive youth, was strong and created an important network to support ART adherence; 4) the burden of taking multiple medications daily frustrated youth, often leading to so-called ‘drug holidays.’ Adolescent and youth-specific issues around disclosure emerged across three of the four barriers.

**Conclusions:**

To be effective, programs and policies to improve ART adherence among youth in Uganda must address the special challenges that adolescents and young adults confront in achieving optimal adherence. For example, training on budgeting and savings practices could help promote their transition to financial independence. School staff could develop strategies to help students take their medications consistently and confidentially. While challenging to extend the range of services provided by HIV clinics, successful efforts will require engaging the family, peers, and larger community of health and educational providers to support adolescents and young adults living with HIV to live longer and healthier lives.

**Trial registration:**

ClinicalTrials.gov Identifier: NCT02514356. Registered August 3, 2015.

## Background

Over 1.3 million Ugandans are living with HIV; prevalence among individuals aged 15–49 currently stands at 5.9% [1]. Of note, those 10–24 years of age comprise 33% of the population, but account for nearly 50% of the country’s HIV/AIDS cases [2]. “Pre-ART” co-trimaxozole (henceforth ‘prophylaxis’) and ART have improved the life expectancy of people living with HIV/AIDS in Uganda dramatically, and ART scale-up has resulted in over 72% of these Ugandans receiving ART [1, 3–8]. However, the success of these drugs is highly dependent on adherence to medication and retention in care to slow the progression to AIDS; lengthen survival; sustain viral suppression; and prevent drug resistance and loss of treatment options [9–14].

Research from resource-poor settings documents how adolescents and youth have lower medication adherence than adults [15–17]. A 2015 comprehensive review of studies focused on adolescents in sub-Saharan Africa (SSA) identified multiple levels of barriers impacting ART adherence [16]. These included sociodemographic factors (e.g., poorer adherence among older adolescents as well as those living in spaces with less privacy such as foster care or orphanages); structural and economic factors (e.g., limited access to food, high cost of transportation, political instability further limiting access to HIV care); psychosocial factors (e.g., limited caregiver supervision, small support networks); individual factors (e.g., forgetfulness); treatment-related factors (e.g., high pill burden, negative side effects, challenging transition between pediatric and adult HIV treatment services); and individual resilience factors (e.g., good adaptive skills and positive expectations for their future were associated with better adherence). A 2018 systematic review of studies focused on adolescents in SSA reported similar findings, identifying stigma, ART side-effects, lack of assistance, and forgetfulness as important barriers; facilitators included caregiver and peer support, and youth having knowledge of their HIV status [17].

Few studies from Uganda report youth adherence to ART or prophylaxis, and some studies indicate serious adherence problems [18–22] . Some of the barriers noted included treatment holidays (i.e., breaks in ART adherence); delays in disclosure of HIV status by caretakers; stigma, especially in boarding schools; diminishing or lack of clinical support [20]; and living in rural areas [19]. A study from one of our partner clinics [18] and our own study [21] among HIV positive youth in Uganda showed that 71–74% of participants did not reach clinically meaningful levels of adherence. Similar levels of poor adherence have been noted in other studies focused on adolescents and youth in Uganda [22] and other SSA countries [23].

Such studies highlight the urgent need to better identify the adolescent and youth-specific barriers to ART adherence in Uganda. Therefore, we qualitatively explored barriers to ART adherence in Uganda among adolescents (age 14–17) and youth (age 18–24), from their own perspective, complemented with insight from community members and healthcare providers, to better understand what factors especially complicate ART adherence for these groups.

## Methods

As part of a larger randomized controlled trial (RCT) [21, 24], we conducted focus groups (*n* = 7) in March and April 2015 with key stakeholders to elicit information on a range of topics related to HIV among adolescents and youth in Uganda. For this article, we focus on a component of the larger qualitative dataset to understand barriers to ART adherence specific to these groups.

### Study population

The sample included community advisory board members (CAB); healthcare providers (henceforth “providers”); and patients registered with an urban primary HIV clinic in Kampala, Uganda, an area that is largely representative of other areas in the capital and includes lower-income market areas as well as upscale shopping areas.

### Recruitment of study participants

We conducted focus groups with several different types of participants including the CAB, providers, and adolescents and young adults (Table 1). The CAB included standing members who meet on a regular basis to discuss clinic related issues. We were able to join a regular CAB meeting and use some of the meeting time for a focus group. The CAB included members from all aspects of the community including church leaders, formally elected community councilmen, medical and other providers (external to the HIV clinic), and persons living with HIV.

**Table 1:** Focus groups at the HIV clinic in Kampala, Uganda

Participant Type	Eligibility Criteria^a^	Participants
Community Advisory Board (*n* = 1)	Requested attendance from existing CAB members.	9 participants: Community representatives working with the HIV clinic. They had diverse tasks including identifying and introducing new clients to HIV treatment and conducting home visits to support ART adherence.
Providers (*n* = 2)	Requested participation from a range of different providers with frequent client contact.	Group A: 1 patient advocate, 2 clinic managers, 2 research managers, 1 psychologist, 1 dispensing staff, 1 receptionist Group B: 5 counselors and 2 dispensing staff
Adolescents and Youth (*n* = 4)	Primary eligibility confirmed by medical records: HIV-positive; age 14–24; on ART or co-trimoxazole; engaged in HIV care for at least 3 months; not currently pregnant or having any opportunistic infections such as TB. Additional eligibility confirmed by self-report: If a minor, must have disclosed HIV status to a caretaker; intention to stay at the clinic for the following year; owned a phone at the time of the study or had regular access to one (at least one hour per day, five days a week); not currently participating in another health-related study. Exclusion criteria: Boarding school attendance (as they do not allow phone usage).	Group A with youth boys 18+: 8 boys Group B with adolescent boys < 18: 6 boys Group C with youth girls 18+: 6 girls Group D with adolescent girls < 18: 5 girls

^a^Eligibility criteria for the focus groups were the same used for the larger parent study [21, 24], of which these FGs were a subcomponent

We purposively sampled providers to reflect different types (such as clinic director, physician, nurse, counselor) and different dimensions of service (pharmacy, adherence counseling). We conducted separate focus groups for youth aged 18 and older, and for those younger than 18. Characteristics of each focus group are summarized in Table 1.

To recruit patients, clinic staff reviewed clinical records and the electronic databases to identify eligible clients among those in attendance at the clinic during the day of the focus group. Clinic staff provided the study coordinator with a list of eligible clients, from which the study coordinator randomly selected 6–8 clients for participation and obtained verbal consent (as requested by the study IRBs) from respondents interested in participating in the study. During the consent process, trained recruiters emphasized repeatedly that participation was voluntary, and that the same level of services would be provided irrespective of whether the patient approached decided to participate or not. Consistent with incentives provided by previous research studies conducted at the same clinic, CAB members and providers were given the equivalent of $16 USD for their participation and adolescents and youth received reimbursement of about $8 USD. All participants were also given lunch, a snack, and transportation money to the focus group.

### Focus group guide

We created a semi-structured focus group guide based on our review of the peer-reviewed literature, in combination with our extensive experience working with HIV-infected youth in Uganda and elsewhere [21, 25, 26] . We elicited information about barriers to ART adherence and about adolescents and youths’ support systems (e.g., family and friends). We created open-ended questions to generate a range of responses.

### Data collection

Facilitators for these focus groups had been hired to conduct the qualitative research components of the parent study [21, 24]. Both facilitators had previous research experience focused on ART adherence at the HIV clinic where the focus groups were conducted. Facilitators were provided a focus group guide depending on the group of participants being interviewed. The focus group discussions were audiotaped, translated from Luganda into English and transcribed verbatim, with names or other identifying information omitted.

### Data analysis

We used a directed content analysis [27]. This process started with uploading data into Dedoose software [28]. Two interviewers (SM and UZ) independently reviewed each transcript and developed a structured codebook to identify a priori identified and emerging themes. As is standard with a directed content analysis, the initial set of themes was informed by existing issues identified in the peer-reviewed literature, complemented by our collective experiences with ART adherence in resource-poor settings. After an initial review of the transcripts, we further revised the codebook during joint coding of two subsequent focus group transcripts. We added examples to the coded themes, when needed, to ensure reliability of coding across interviews. We determined inter-rater reliability using the Cohen’s Kappa coefficient for a randomly selected set of transcript excerpts. Based on our values of a pooled Cohen’s Kappa of 0.79 and 0.80 across the two reviewers indicating good agreement, we single-coded the rest of the interviews. The two coders met weekly to resolve coding and interpretation differences, and to identify emergent themes and relevant exemplars. Data that did not appear to fit into existing codes were discussed to determine if they represented a new category or a subcategory of an existing theme.

To further draw meaning from the content of the organized quotes, the research team collectively discussed themes, identified the range of responses within each theme, and also discussed the relationship between themes. The research team also returned to the organized quotes to discuss the difference between patterns or themes that were directly expressed in the text (e.g., pill burden) and those derived through analysis (e.g., the role of HIV-related stigma).

Finally, we ensured that the quotes included in the results represented all voices from the focus groups, acknowledging that certain issues may be better articulated by some groups based on their range of experiences (e.g., CAB members and providers), while other issues are best articulated from the participants themselves (e.g., adolescents and youth).

The study was approved by the RAND Human Subjects Protection Committee, the HIV clinic’s IRB, and the Uganda National Council for Science and Technology.

## Results

We identified four barriers to ART adherence and noted how issues around disclosure cut across three of the barriers (Barriers 2, 3, and 4). The characteristics of each focus group are detailed in Table 1.

### BARRIER 1 - poverty had immediate and longer-term impact on ART adherence, especially in child-headed households

Both youth and providers noted how poverty made it difficult for adolescents to purchase food, complicating their ART adherence:

*“… this medicine that we swallow, one of the [things] you are instructed [is] that you must eat thirty minutes before you take that drug. But then you find in that time, most times, there is nothing to eat, and so what do you do before you start, before you swallow medicine, and yet your time has approached? That also is a challenge we face as youth.” – Youth boy age 18 and older.*

This issue was further exacerbated for youth living in child-headed households, as articulated by the following provider:

*“Then there is also an issue of these children living in child-headed families and they have to cater for themselves, so it challenges them at times… maybe they don’t have food, they don’t have water to drink yet… we always encourage them that before you take your medicine, eat something, even if it’s just a cup of porridge or banana. But there are times when they cannot actually access it. So, it all goes around the poverty levels that are in most of the household.” – Provider.*

Subsequent interviews suggested that child-headed households were common because many HIV-positive adolescents and youth had lost their biological parents to HIV.

While the transition from adolescence to adulthood was often difficult in general, the additional responsibility of establishing a steady income to purchase food was especially stressful:

*“… And these adolescents here we are preparing them to live independent, [but they say], ‘My parents are not here, [so] how am I going to live now that am 18 - what should I do?’”– Provider.*

### BARRIER 2 – School settings presented additional complications

Respondents identified issues around storing and remembering to take ART while at school:

*“The other problem we face, like me, I don’t spend time in one place. Every time I am moving, let me say, now am a student, you find that most of the time I am at school, but, to pack my drugs and make sure I reach at school, but to make sure that the drug is still safe to be able to swallow is hard for me. So, in the time to swallow medicine I find that am cautious about taking the drug which appears like spoilt, it might bring problems for me and so I leave it home and I find that I don’t swallow medicine in time.” – Youth boy age 18 and older.*

Further, even when they remembered to take ART, youth identified special challenges associated with school-specific tasks (e.g., taking an exam):

*“Also, during examination time, like maybe you are starting the paper at 9 am and you are supposed to take your drugs at 10 am and you find yourself [in the middle of] a paper. You can’t give someone a reason that am going out to take your medicine.” – Youth girl age 18 and older.*

Also noted was the lack of support among staff at school. One adolescent boy said:

*“…sometimes we also lack advice where we are like at school whenever we are there we don’t have people to come and tell us what to do, the only advice we get is from here [the HIV clinic].” Adolescent boy below age 18.*

Participants reported how the act of taking a pill in a school setting, surrounded by their friends, could challenge their ability to keep their HIV status confidential. A youth explained:

*“…you take your medicine at school, in the dormitory… sometimes you don’t want your fellow students to know that you are infected because when they know [you are infected with HIV] they are going to … start talking about you and whenever they talk about you in [school], you will feel shy….you feel uncomfortable in the class…” - Youth boy age 18 and older.*

While adolescents and youth in our sample did not attend boarding school at the time of data collection, one adolescent boy spoke about his previous experience in a boarding school. He relayed how maintaining privacy regarding his medication could be especially complicated in this setting due to the fact that there can be even less privacy for students in shared living quarters:

*“But when you are in boarding [school], you have your suitcase there and your drugs are in there but you can’t get them out because your colleagues are around you… [when] then they find out that it’s a tin then they start suspecting and isolating you.” Adolescent boy below age 18.*

This quote also speaks to the perceived risk of disclosure in a school setting: just taking medication can act as a public marker of HIV status.

### BARRIER 3 – Family and friends affect ART adherence in different ways

Frequently, respondents noted that the influence of family was inconsistent. The following quote relays how one youth often felt he was on his own:

*“…but most times, we don’t have people to help us, to guide us take our medicine, it’s me.”* - *Youth boy age 18 and older.*

One provider explained how a youth’s family structure can be quite variable.

*“… different youth will have different people to care for them. There are those that all my parents died but I have an aunt at home, but this aunt is very active. Then there is this who is like at home I stay with my grandmother, but she is never at home or I stay with these people, but they are never at home, so sometimes I find myself when am alone. So, it depends, different people behave differently in their homes.” – Provider.*

As previously mentioned, many of the youth had HIV-positive parents who died and the youth reported an ongoing struggle with their loss. One youth expressed how common the loss of a parent is and specifically, how the loss continued to impact them throughout their life:

*“…when you don’t have a mother [or] you don’t have a dad, life is very, very difficult… Since I was born I have never seen my mum [or] my dad… in my heart [I know] that if mum was around, she would have [helped] me.”* - *Youth boy age 18 and older.*

Without their biological parents, youth faced a constant change in guardianship, challenging their ability to grow up in a stable household:

*“Then there is also another issue of change of guardians. Today this adolescent is [with their] aunt so and so, tomorrow … at the grandparent's and the other day is taken by the uncle. So, it affects [them] because not all these people around him actually know that he’s supposed to take his medication. Sometimes when he’s shifting from the auntie’s place to the granny’s [place], he leaves the drugs at the auntie’s. So, while at the grandparent’s house, he doesn’t actually take his medication and it really affects their adherence especially when the parents have died and they are taken on by their guardians.” - Provider.*

Across all interviews, peer influence between HIV-positive youth was highlighted as an important factor supporting ART adherence.

*“So, I realized they work together, there are groups which really work together to ensure that they take their drugs well but there are other groups also which discourage their colleagues… yet somehow they discourage them, and it affects their adherence so peer pressure also influences them so much.” – Provider.*

One youth described how one of his HIV-positive friends encouraged him to continue taking his medication when he struggled to adhere:

*“Well from the first day I met him is the day we both started medication here and became friends. I asked him how the drugs treat him, and he told me that it didn’t treat him so bad apart from the dizziness, but for me, the drugs treated me badly, whenever I swallowed a pill, I would vomit, get a skin rash and every time I would go to the bathroom with diarrhea. He told me not to worry that I would be fine with time but encouraged me not to stop taking the drugs, and so I think if I had stopped swallowing the drugs, I would not be here now*.” - *Adolescent boy below age 18.*

But friendships often extend beyond their focus on ART adherence, as one young female noted. While they do sometimes talk about their ART medications and check in with each other, it often becomes a side conversation to talking about their social lives. This participant simply said:

*“We basically talk about social life.” Adolescent girl below age 18.*

With respect to disclosure, several providers relayed previous experiences with adolescent patients who had not been told that they were born with HIV, complicating efforts to underscore the critical importance of ART adherence:

*“…some of these adolescents don’t know why they should take this medication. We have interacted with some who think or have been told by their parents and guardians that they have heart disease or maybe kidney disease and that’s why they are taking. So, as they grow, they get fatigue and they are like, ‘Why doesn’t this heart heal? Why am I continuing to take [it]?’ I interacted with a 24-year old who was tired and was like, ‘I think I’m tired because this heart is not healing.’ So, non-disclosure affects their adherence” - Provider.*

### BARRIER 4 - the pill burden led some to take so-called ‘drug holidays

Participants noted the challenge of having to take multiple pills, instead of one combined pill:

***“*** *For me the problem I face, I would request and earnestly request, if possible, to change for us the drugs which we swallow twice in a day, because when I swallow the first time in the day I am alone. Sometimes when I come back to swallow the second dose, I have visitors, because I am a youth, I have visitors, I have a room, they come to watch TV and so on…So now they are in my time when am supposed to be swallowing medicine, that’s the problem I face and so I wanted it to be a one-time dose.” Youth boy age 18 and older.*

Several of the participants were born with HIV, and both the providers and the youth referenced the exhaustion they felt from the long-term commitment to ART. Providers reported that the pill burden, and the associated fatigue of dealing with their HIV status, caused some youth to take extended breaks or so-called ‘drug holidays’:

***“*** *… we have this drug holiday issue whereby they have a policy of saying ‘I have taken these drugs for so long, let me give myself a drug holiday, for this month I won’t take [it].’” - Provider.*

Disclosure posed an issue with respect to pill burden: youth believed taking ART often served as a public marker of HIV status. Specifically, respondents noted how youth taking ART can feel as though it forced them to disclose their HIV-positive status:

*“..what makes some youth hide themselves when taking drugs is that they think that when they take the drugs publicly if he’s a boy he might fail to get a wife and if she’s a girl she thinks she will fail to get a man to marry her yet they also have their worldly desires…”.–Community Advisory Board Member.*

Another adolescent girl went on to say that it is not only the fear of disclosure, but that they’ve had experiences with people spreading rumors about their HIV status once they have been seen taking pills. She said:

*“If you swallow these drugs where there are people; like me, there is a lady who went on telling everyone that am positive, I don’t know how she got to know but finally the people around me started isolating me that I will infect them. So, the people around us make us isolate ourselves.” Adolescent girl below age 18.*

## Discussion

Four barriers to ART adherence emerged: 1) Poverty limited youths’ ability to purchase food, making it difficult to avoid the side effects of taking ART on an empty stomach; poverty also complicated efforts to become economically independent in their transition from adolescence to adulthood; 2) The lack of privacy in school schedules complicated ART adherence; 3) family support was unreliable, and youth often struggled with a constant change in guardianship because they had lost their biological parents to HIV. In contrast, peer influence, especially among HIV-positive youth, was strong and created an important network to support ART adherence; 4) The pill burden associated with HIV treatment frustrated youth, constantly reminding them of their HIV status and often leading them to taking so-called ‘drug holidays.’ Youth-specific issues around disclosure emerged across three of the four barriers.

The impact of poverty in SSA, and many of the costs associated with ART adherence, have been increasingly documented in the peer reviewed literature [29–32]. For example, several multi-country studies that included Uganda, documented how, despite free provision of ART, other related costs (e.g., transportation, lost wages due to time spent seeking healthcare) posed a significant barrier to ART adherence [30, 31]. Food insecurity, and its impact on ART adherence, is associated with poor HIV outcomes (e.g., incomplete viral suppression and low CD4 count) in resource-poor settings [33–35] and in Uganda specifically [36]. It is likely that the financial transition from adolescence to adulthood places an additional burden on older adolescents on the cusp of this change. While significant research has focused on the clinical transition from pediatric to adult HIV care in SSA [37, 38], limited research has focused on the implications of this transition outside of the clinical context.

Adolescents and youth noted barriers to ART adherence associated with different aspects of school settings. These findings are consistent with existing studies. A comprehensive review of ART adherence challenges in SSA noted that adolescents living in boarding schools, foster care, or orphanages were often faced with a lack of privacy, lack of support, or stigma if they were discovered to be using ARTs, which made it difficult to maintain medication use [16]. Studies in resource-poor settings have noted how for many, the home provides a safe space to manage ART adherence [39] while schools can pose special challenges. In Uganda specifically, two qualitative studies reported that the stigma and discrimination experienced by HIV-positive youth were mainly experienced in boarding schools and highlighted the need for focused programs to address these barriers to ART adherence [20, 40].

Respondents from all focus groups noted how the influence of family was inconsistent and often further complicated by the frequent change in guardianship resulting from the loss of biological parents to HIV. The role of family and its impact on ART adherence has been documented in both high- [41] and low-resource settings [32, 39]. Several studies note the stress experienced by caregivers, who often feel ill-prepared to provide the support needed by their HIV-positive youth [42]. A specific focus on shifts in guardianship has been noted among studies on youth orphaned by HIV in SSA [43, 44] and Uganda [45]. It is possible that compared to infants and young children, adolescents and youth are seen as more independent and are therefore less likely to receive the support needed as they transition towards adulthood. While the basic needs are indeed different, the developmental needs of these groups are still significant and warrant attention [46].

Respondents also noted that peer influence between HIV-positive youth was strong and created an important network to support ART adherence. One study in South Africa explored the relationship between the level of family functioning and the effectiveness of peer-based interventions to address ART adherence. The study revealed that peer support for adherence had a positive effect on immunological restoration in functioning families and had a negative effect in dysfunctional families [47]. Similar studies indicate that attention to the family dynamics, rather than simply engaging all families, and building networks of support among HIV-positive peers, may help improve ART adherence for older adolescents going forward [48, 49].

Problems associated with HIV-related pill burden raised by our study respondents are consistent with findings from other studies in SSA. Pill burden can have many different dimensions including the quantity [19], the taste [20], and the challenge of taking pills across different social settings [32]. In our study, respondents specifically spoke to the challenge of taking multiple pills as well as the difficulty of having to take pills daily, further illustrating the multi-dimensional challenges associated with the simple act of taking a medication. Often the pill burden associated with HIV was raised in the context of serving as a constant reminder of an individual’s own HIV status [50]. As noted by respondents, it can sometime lead to youth taking breaks in their treatment [20] or ‘drug holidays,’ which significantly increases the likelihood of developing drug resistance to their existing ART regime and requiring far more complex and costly ART regimes [20].

Going forward, some biomedical advances that are increasingly but not consistently available in Uganda may address some of the challenges associated with pill burden. These include reduction in the number of pills required [51], and reduction in the required frequency of dosages. Long-acting injectable ART medications shift the daily pill taking responsibility to receipt of an injection every three months [52]. However, there is often a lag time before such treatment advances are successfully implemented for adolescents and youth, given the challenges associated with testing the safety and efficacy of biomedical strategies in younger populations [53].

Disclosure of HIV status exacerbated the impact of several barriers. Specifically, according to our respondents, disclosure of HIV status was complicated in school settings. Several of the aforementioned studies focusing on issues faced by HIV-positive youth in school settings also noted that taking medication was particularly challenging in physical spaces with limited privacy [50]. With respect to friends and family, challenges with disclosure were noted by providers as they had previous experience with youth who were in treatment but still had not been told that they were HIV positive. When and how best to tell youth that they are HIV-positive [54, 55] has been well documented in SSA. Some studies have shown that disclosure of HIV can improve ART adherence [56, 57]; however, a systematic review reported conflicting results [58]. Of note, since all adolescents and youth in our study knew their HIV status as an eligibility requirement of participation in the parent study, this issue was not explored in great depth in our FGs, but it certainly warrants further attention. Another issue consistently raised in other studies but not flagged here was the frequency with which adolescents and youth must chose to disclose – or not – their HIV status. Mainly, the ebb and flow of friendships and sexual partners [59] can require constant decision making about disclosure, which can be very different from those of adults with more stable, longer-term relationships (e.g., spouse). Finally, another way in which HIV disclosure complicated existing barriers relates to pill burden: for many, having to take medication served as a public marker of HIV status. Taken together, we see how disclosure intersects with the physical location of youth (e.g., schools) and with their personal relationships (e.g., friends and family). Such complications require them to constantly negotiate their daily responsibilities of taking their ART medication.

While respondents were not directly probed on the role of stigma, it often served as the unspoken reason driving their need to maintain their HIV status private. Across respondents, fear of being ‘outed’ was consistently noted. Despite the substantial progress in shifting HIV from an immediate death sentence to a long-term chronic disease, being identified as HIV-positive still carries substantial social consequences. While the role of stigma especially among youth is well documented [60], Adejumo and colleagues made a particularly nuanced point about the role of stigma for youth [16]. In their comprehensive review of ART barriers, they noted how ART adherence can be a paradoxical source of stigma, as multi-country studies from SSA [61], and from Uganda [62] have found an increase in perceived stigma among adults taking ART, compared to those not taking ART. While there are certainly other studies with contrary findings [63], Adejumo and colleagues highlight how the developmentally appropriate desire to ‘fit in’ may exaggerate anything that differentiates them from their peers. Thus, while the ART medication provides them the health and wellness to ‘feel normal,’ and fit in on some domains, it simultaneously distinguishes them in others. While respondents did not explicitly raise the role of stigma often, the few who did, used particularly powerful words to articulate the pervasive impact of HIV-related stigma in their daily lives. We encourage further work to understand the complex role of stigma in the lives of HIV-positive adolescents and youth.

### Limitations & strengths

Our study has both limitations and strengths. We did not recruit a representative sample; thus study results cannot be used to draw conclusions about hypothesis testing or population-level dynamics. Additionally, we do not have individual-level sociodemographic characteristics on participants nor do we have information to determine route of transmission. From discussions with providers and the study coordinators, it seems that the majority of adolescents were perinatally infected. Some anecdotal evidence suggests that some youth (in particular girls) acquired HIV behaviorally; however, we are unable to confirm this point. Further, while providers and CAB member referenced the additional barriers introduced by attending boarding school, few adolescents in the study could speak to these issues as a result of the fact that those currently attending boarding school were excluded from the study due to criteria from the larger RCT of which these FGs were a component. However, youth did relay facing many of the issues raised in a school context, though the ways in which these issues differ in boarding schools could not be discussed extensively from the youth’s perspectives. These limitations should be considered in light of the study’s strengths. We included a diversity of perspectives (e.g., CAB, providers, as well as adolescents and youth themselves) and build on existing literature regarding how adolescents and youth in resource poor settings confront unique barriers to ART adherence.

## Conclusions

Our results highlight special needs for future public health programs and policies meant to address barriers to ART adherence among older adolescents. For example, with respect to the impact of poverty, our findings underscore the need to address known costs (e.g., providing transportation and food vouchers) as well as to consider if older adolescents need training (e.g., how to budget and save money) to support themselves as they transition towards financial independence.

Regarding barriers presented by schools, the Ugandan Ministry of Education has taken concrete steps to establish guidelines and school-based support systems for HIV-positive youth [64]; however, a comprehensive analysis of issues faced by HIV-positive youth in Uganda also suggested the need to ensure that existing policies are consistently implemented to improve HIV-related support services for students [65]. Our findings also suggest that public health programs and policies should give special attention to how constant changes in guardianship might complicate ART adherence in the context of the biological and social transitions experienced by older adolescents.

With respect to pill burden, even as biomedical advances may reduce the number and frequency of pills required, and diminish some related concerns, youth should be prepared with a broader toolkit of strategies to navigate ongoing challenges. For example, proactively counseling adolescents and youth on things they can say to excuse themselves (i.e., go to the bathroom), or providing alternative ways to talk about their ART medication if they don’t want to disclose their HIV status, can better enable ART adherence.

Finally, the issues related to disclosure highlight the importance of expanding public health programs and policies to include concrete strategies that help youth navigate these complex social situations. Older adolescents and youth in particular, as they become sexually active and face the added responsibility of disclosing their HIV status to their partners, need specific skills to help them cope with these new and often complicated social and sexual dynamics. Taken together, our study underscores how careful attention is needed to concretely address the special barriers faced by HIV+ adolescents and youth to support them in their transition to adulthood.

## Abbreviations

CAB: Community Advisory Board
FG: Focus Group
HIV: Human Immunodeficiency Virus
RCT: Randomized Control Trial
SSA: Sub-Saharan Africa
TASO: The AIDS Support Organization of Uganda
